# Effects of Platelet Lysate Gels Derived from Different Blood Sources on Oral Mucosal Wound Healing: An In Vitro Study

**DOI:** 10.3390/gels9040343

**Published:** 2023-04-17

**Authors:** Sook-Luan Ng, Nur Ain Azhar, Siti Balkis Budin, Norliwati Ibrahim, Nur Azurah Abdul Ghani, Norzana Abd Ghafar, Jia-Xian Law

**Affiliations:** 1 Department of Craniofacial Diagnostics and Biosciences, Faculty of Dentistry, Universiti Kebangsaan Malaysia, Jalan Raja Muda Abdul Aziz, Kuala Lumpur 50300, Malaysia; norliibrahim@ukm.edu.my; 2 Centre for Diagnostic, Therapeutic and Investigative Studies, Faculty of Health Sciences, Universiti Kebangsaan Malaysia, Jalan Raja Muda Abdul Aziz, Kuala Lumpur 50300, Malaysiabalkis@ukm.edu.my (S.B.B.); 3 Department of Obstetrics and Gynaecology, Faculty of Medicine, Universiti Kebangsaan Malaysia, Jalan Yaacob Latif, Bandar Tun Razak, Cheras, Kuala Lumpur 56000, Malaysia; nurazurahag@gmail.com; 4 Department of Anatomy, Faculty of Medicine, Universiti Kebangsaan Malaysia, Jalan Yaacob Latif, Bandar Tun Razak, Cheras, Kuala Lumpur 56000, Malaysia; norzana@ukm.edu.my; 5 Centre for Tissue Engineering and Regenerative Medicine, Faculty of Medicine, Universiti Kebangsaan Malaysia, Jalan Yaacob Latif, Bandar Tun Razak, Cheras, Kuala Lumpur 56000, Malaysia

**Keywords:** cord blood, peripheral blood, human platelet lysate gel, oral ulcer, wound healing

## Abstract

The rapid healing of oral ulcers is important to prevent secondary infection, especially for chronic oral ulcers. Platelet lysate (PL) is rich in growth factors for cell growth and promotes tissue regeneration. Hence, this study was performed to compare the effects of PL originating from umbilical cord blood (CB) and peripheral blood (PB) on oral mucosal wound healing. The PLs were molded into gel form in the culture insert with the addition of calcium chloride and conditioned medium for sustained release of growth factors. The CB-PL and PB-PL gels were found to degrade slowly in culture and their degradation percentages by weight were 5.28 ± 0.72% and 9.55 ± 1.82% respectively. The results from the scratch assay and Alamar blue assay showed that the CB-PL and PB-PL gels increased the proliferation (148 ± 3% and 149 ± 3%) and wound closure (94.17 ± 1.77% and 92.75 ± 1.80%) of oral mucosal fibroblasts compared to the control with no statistical differences between the two gels, respectively. Quantitative RT-PCR showed that mRNA expressions of collagen-I, collagen-III, fibronectin, and elastin genes in cells treated with CB-PL (11-, 7-, 2-, and 7-fold) and PB-PL (17-, 14-, 3-, and 7-fold) decreased compared with the control, respectively. The concentration of platelet-derived growth factor of PB-PL gel (1303.10 ± 343.96 pg/mL) showed a higher trend than CB-PL gel did (905.48 ± 69.65 pg/mL) from ELISA measurement. In summary, CB-PL gel is as effective as PB-PL gel in supporting oral mucosal wound healing, making it a potential new source of PL for regenerative treatment.

## 1. Introduction

Oral ulcers are defined as damage to the epithelium and lamina propria of the oral mucosa [1]. There are several factors that can lead to oral ulcers such as infections, immune-related conditions, physical or chemical trauma, and neoplasia [2]. Wound healing is a normal physiological reaction to restore the integrity and structure of injured tissue to its normal state. Wound healing is a complex process that can be separated into four overlapped phases, i.e., hemostasis, inflammation, proliferation, and remodeling [3]. Homeostasis is a state where fibrin binds to platelets and cross-links to form a clot to stop the bleeding, which is then followed by an inflammatory process that involves activation of immune cells and the production of proinflammatory cytokines that lead to the recruitment of epithelial cells, fibroblasts, and endothelial cells. Next is the proliferative phase that involves fibroblast proliferation and differentiation to myofibroblasts, collagen deposition, granulation tissue formation, angiogenesis, and re-epithelization. During the tissue remodeling phase, excessive extracellular matrix (ECM) will be broken down and the remainder will be remodeled to raise the tensile strength of the wound [4,5].

Fibronectin, elastin, collagen-I, and collagen-III are significant biomarkers in the wound healing process. Fibronectin is deposited in abundance during wound healing to improve the function of the cells besides promoting angiogenesis, decreasing inflammation, and modulating collagen deposition and degradation. Fibronectin is an essential component that improves healing quality by preventing scar formation through collagen degradation during the remodeling phase [6]. Elastin is also an important biomarker in wound healing, which is responsible for tissue elasticity and provides mechanical strength to accelerate wound closure. The lack of elastin may contribute to scar tissue formation [7].

Collagen-I is another biomarker of wound healing that can be found in the oral cavity. Collagen-I makes up 80–85% of the skin ECM compared to collagen-III, which only constitutes 8–11% of the ECM [8]. Collagen production plays an important role in determining the quality of tissue regeneration, structural integrity of the healing tissue, and matrix remodeling. Collagen-III is highly synthesized during the early phases of wound healing and is gradually replaced by collagen-I [5]. Collagen-III provides the support needed for the growth of new capillaries and provides for increased fibroblastic activity during the proliferative phase. It also accelerates the rate of wound healing by forming a network that can support cell adhesion and tissue integrity. Therefore, insufficient collagen deposition or excessive collagen degradation during the wound healing process can interrupt the wound closure and lead to the development of chronic wounds [4].

Despite receiving proper wound management, certain oral ulcers could still progress to chronic wounds that require a healing period longer than 12 weeks, which increases the risk of bacterial infection and interrupts the normal wound healing process. Thus, regenerative medicine has been employed to overcome this problem. One of the approaches is the use of peripheral blood-platelet lysate (PB-PL), which has been applied in certain medical fields such as dentistry and oral implantation, ophthalmology, sports medicine, cosmetics and dermatology, and the treatment of ulcers and soft tissue defects [9]. PB-PL is rich in growth factors that can be found in α-granules of adult blood platelets, which include vascular endothelial growth factor (VEGF), platelet derived growth factor (PDGF), fibroblast growth factor (FGF), transforming growth factor-beta (TGF-β), and insulin-like growth factor 1 (IGF-1) [10,11]. Previous research has shown that PB-PL can accelerate the proliferation of cultured cells compared to fetal blood serum (FBS) in supporting the cell culture by increasing mRNA regulation for proto-oncogene protein transcription factors, sex-determining region Y, and Kruppel-like factor 4 [12]. Besides that, PB-PL is also capable of stimulating various factors that are important in ECM formation during the wound healing process [13,14]. Although autologous usage of PB-PL gives various benefits in the wound healing process, its clinical use has been controversial regarding its quality that differs among patients, influenced by the patient’s physiological state. Besides that, the collection of blood in large quantities to obtain autologous platelet-rich plasma (PRP) will also add an additional health burden on patients.

Cord blood-platelet lysate (CB-PL) is a potential alternative treatment to replace the use of autologous blood. CB-PL is extremely useful in cases of contraindications to the production of autologous platelet derivatives due to patient reluctance or discomfort, inappropriate venous access, and the existence of autoimmune, inflammatory, or hematological diseases [9]. This is because CB-PL is known to have a high concentration of growth factor content just like the PB-PL. Orlando and colleagues found that CB-PL has a higher growth factor content such as TGF-β2, FGF, PDGF, VEGF, and nerve growth factor (NGF) compared to PB-PL [9]. Besides that, Losi and colleagues showed that CB-PL is likely to be useful for producing platelet lysate in large quantities and surpassing the standard value for clinical use as it is able to increase cell viability and proliferation compared to basal medium [15]. In addition, CB-PL also showed a higher potential in supporting mesenchymal cell proliferation than PB-PL and FBS [16]. Due to its high growth factor content, CB-PL has been successfully used in the healing of the oral mucosa in patients with epidermolysis bullosa dystrophy [17]. Therefore, CB-PL is rich in growth factors and can support cell functionality, thus making it an attractive alternative to PB-PL for tissue engineering applications [18].

Platelet-derived growth factor (PDGF) released by platelets plays an important role in wound healing. Platelet lysate containing PDGF is effective in expediting wound healing as proven by the rapid migration of fibroblasts to close the wound in the scratch assay [19]. PDGF is also able to stimulate phenotypic changes of fibroblasts to myofibroblasts, which induce contractile forces that lead to wound closure [8]. This study’s aim was to evaluate the effect of CB-PL and PB-PL gels on wound healing of oral mucosal fibroblasts through the determination of wound closure percentage, ECM gene expression, and the release of PDGF-BB. 

## 2. Results and Discussion

### 2.1. Cell Proliferation in PL Gels 

**Figure 1:**
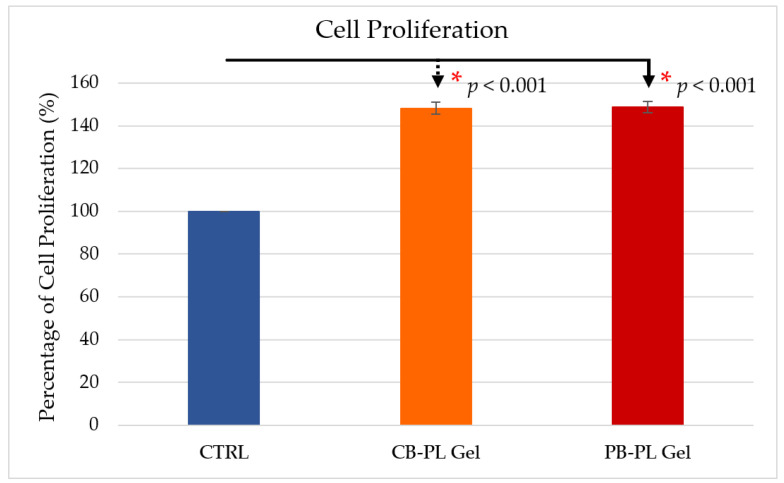
The cell proliferation percentages for CB-PL and PB-PL were computed relative to the serum-free medium/CTRL, which was set as 100% for normalization. The data were reported as means ± SEM of six independent samples (*n* = 6). The statistical significance of CB-PL gel and PB-PL gel in comparison to CTRL was indicated by * *p* < 0.05.

The proliferation percentage of human mucosal fibroblasts (HOMFs) treated with CB-PL gel (148 ± 3%) and PB-PL gel (149 ± 3%) was significantly higher than that of the serum-free control (100%). However, there was no significant difference between the proliferation of HOMFs treated with the CB-PL and PB-PL gels (Figure 1). Similarly, Parazzi and colleagues found that there was no difference between CB-PL and PB-PL in supporting cell proliferation, but both were more effective in supporting cell proliferation compared to FBS [20]. This could possibly be due to CB-PL and PB-PL displaying similar bioactive molecular profiles where both have higher concentrations of pro-inflammatory cytokines and anti-inflammatory factors than their corresponding plasma samples [16]. However, this trend of cell proliferation was contrary to the findings of previous studies. The proliferation of mesenchymal stem cells treated with CB-PL was faster than those of PB-PL and FBS due to the higher content of growth factors in CB-PL [21]. The PRP preparation protocol was not standardized for the type and amount of anticoagulant used in blood collection, centrifugation time and speed, storage condition, and platelet activation in the previous studies. The content variation in the platelet derivatives provides different in vitro, in vivo, and clinical outcomes [22]. 

### 2.2. Cell Migration in Wound Healing 

**Figure 2:**
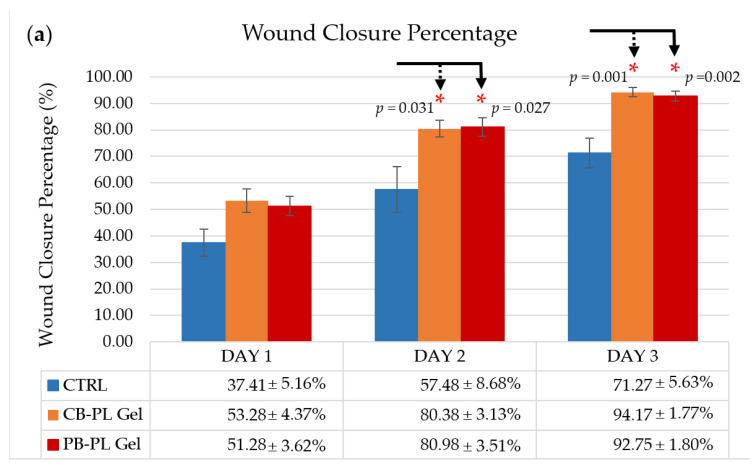

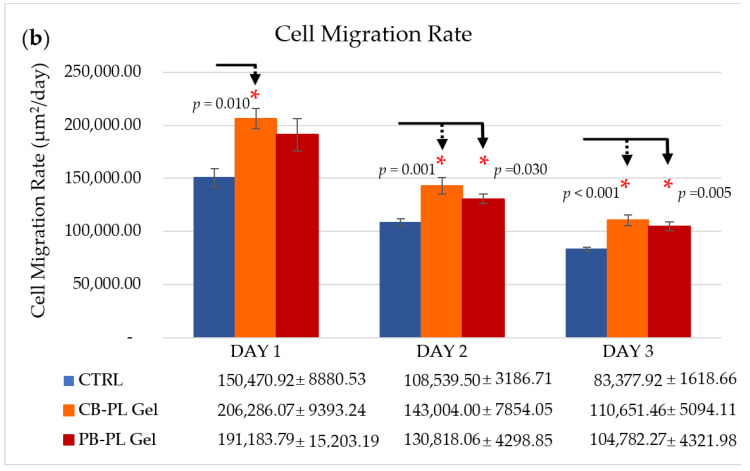
Wound healing effects of CTRL, CB-PL gel, and PB-PL gel on HOMF migration. The wound healing was assessed by measuring the (**a**) wound closure percentage and (**b**) cell migration rate after exposure to PL gels for 1, 2, and 3 days. The results were based on six independent tests (*n* = 6) and the means ± SEM were provided. The statistical significance of CB-PL gel and PB-PL gel in comparison to CTRL was indicated by * *p* < 0.05.

**Figure 3:**
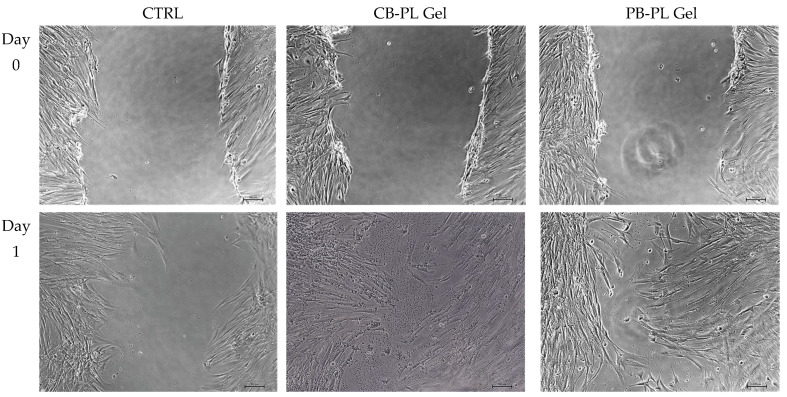

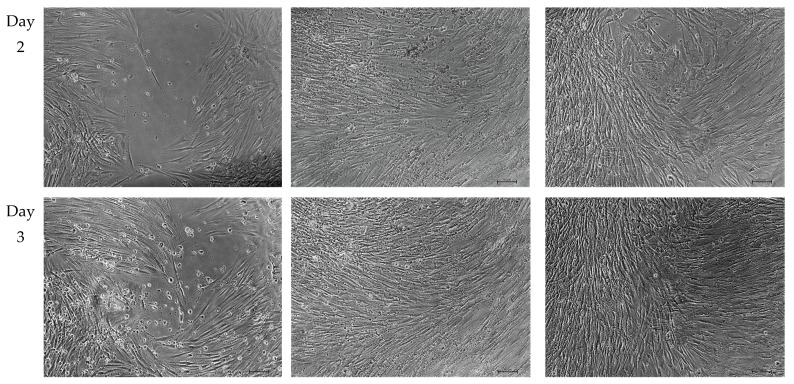
The cell migration of HOMF under treatment of CB-PL gel, PB-PL gel, and CTRL was observed using phase contract micrographs at 100× magnification in the wound scratch assay on days 0, 1, 2, and 3. Both PL gels were found to expedite the wound closure compared to the CTRL.

During the proliferative phase of wound healing, there is a process of replacing the transient fibrin matrix with a new matrix consisting of collagen fibers, fibronectin, and proteoglycans to reconstruct the tissue structures in order to restore their function. The fibroblasts are important during the proliferative phase of wound healing. Fibroblasts migrate into the wound under the influence of various growth factors released by platelets [23]. Consistent with the results of cell proliferation, the percentage of wound closure and the cell migration rate for HOMF in the presence of CB-PL gel and PB-PL gel were higher compared to the control on days 1, 2, and 3 (Figure 2). On day 3, a large wound area could still be seen in the control group, while the wounds were almost completely closed after treatment with the PL gels (Figure 3). The PL gels promoted faster wound closure due to their high amount of PDGF and TGF-β, which can stimulate the cell proliferation, migration, and differentiation [21]. PDGF stimulates chemotaxis and rearranges the actin filament system, which causes cell movement and cell shape changes. The growth factors released from PL promote the cell proliferation, migration, activation, and differentiation in order to stimulate wound healing [24].

### 2.3. Gel Degradation

**Figure 4:**
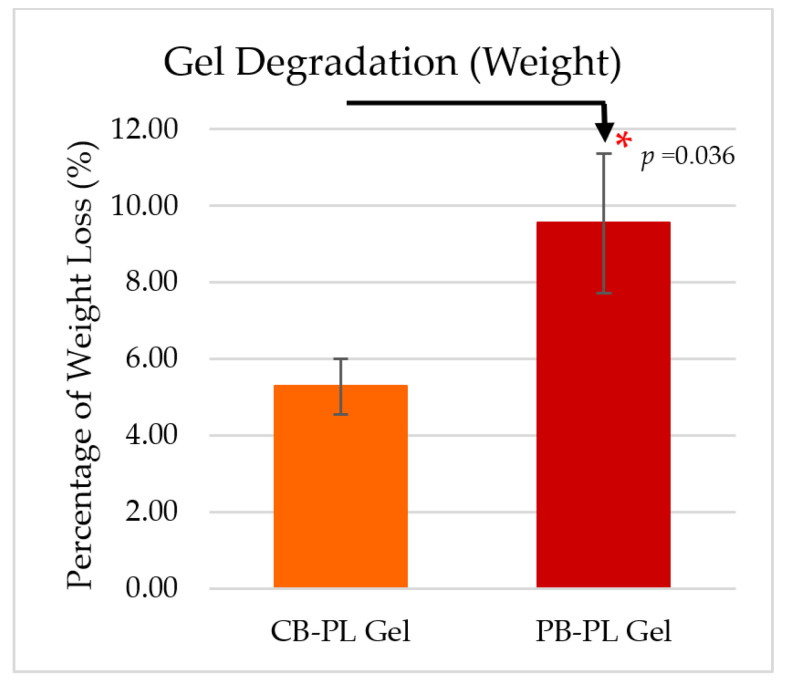
The percentage of gel degradation was evaluated on day 3 based on four independent tests (*n* = 4). The means ± SEM were reported. Statistical significance between PB-PL gel and CB-PL gel was indicated by * *p* < 0.05.

The degradation percentage of PB-PL gel (9.55 ± 1.82%) by weight was significantly higher than that of CB-PL gel (5.28 ± 0.72%) on day 3 (Figure 4). Despite that, the percentage of CB-PL and PB-PL gel degradation over a period of 3 days was still low in this study. PL gels started to liquify after 5 to 7 days submerged in the culture medium. Therefore, this PL gel is likely to be used as a short-term treatment in the future. The application of platelet lysate in gel form is better than liquid form because gel prevents the platelet content from dispersing from the desired site to other parts [25]. Thus, the gel can restrict the movement of molecular content and increase the effectiveness of the treatment.

In addition, the use of platelet lysate in the form of gel is considered an effective method of growth factor delivery because the gel allows the gradual release of its contents. The gradual release of growth factors is highly desirable as it can protect the growth factors with a short half-life and give a sustained therapeutic effect. In this study, PL gel was formed through the addition of calcium chloride (CaCl_2_) to stimulate the conversion of fibrinogen. Fibrin is an alternative platform for growth factor delivery due to its bio-adhesive and biodegradable properties [26]. 

The degradation of CB-PL and PB-PL gels was measured by calculating the percentage of weight loss from the membrane [27]. Previous studies stated that PRP activation involves 2 main processes, which are platelet degranulation to release the growth factor payload of α-granules and fibrinogen cleavage to begin matrix formation. At the end of the coagulation process, the formed platelet gel will restrict the secretion of molecules to the target site [28].

The material used for PRP activation should be chosen according to the release kinetics of PRP to sustain the effect of treatment. The activation material that had been used in this study was CaCl_2_ along with the conditioned medium of HOMF in order to strengthen the physical structure of the gel. The addition of conditioned medium is important because double-centrifuged platelet lysates and PRP usually have a low fibrin density and poor polymerization that will cause the gel to dissolve faster in the culture media. The conditioned medium contains tissue factors and phosphatidylserine, promoting the cross-linking of the PL gel or coagulation [29]. The use of CaCl_2_ in gel polymerization produced a PRP gel with a high concentration of non-activated and functional platelets, thus allowing a constant and gradual release of PDGF-BB. This sustained release of growth factors would be beneficial for wound healing and remodeling [30].

### 2.4. ECM Gene Expression in Wound Remodeling

**Figure 5:**
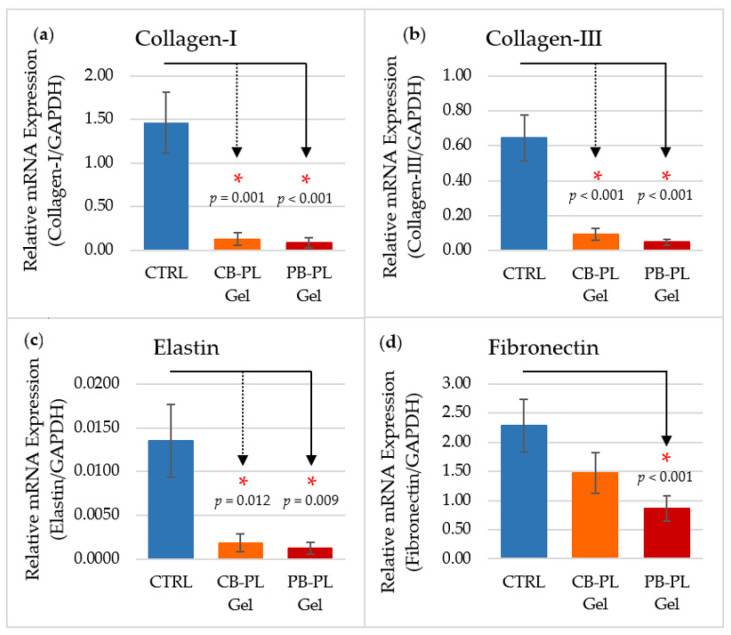
The relative mRNA expression of ECM markers, including (**a**) collagen-I, (**b**) collagen-III, (**c**) elastin, and (**d**) fibronectin, was analyzed among PL gels and CTRL on day 3. The results were based on six independent tests (*n* = 6) and the means ± SEM were reported. The statistical significance of CB-PL gel and PB-PL gel in comparison to CTRL was indicated by * *p* < 0.05.

The gene expression of collagen-I, collagen-III, elastin, and fibronectin of HOMF was significantly lower after treatment with CB-PL and PB-PL gels compared to the control group (Figure 5). The gene expression of ECM markers was presented in means ± SEM (Table 1). There was no significant difference in ECM gene expression between both PL gels. The results of this study are consistent with the literature that reports that PRP treatment reduced the expression of major collagens in tendons, i.e., collagen-I, collagen-III, and elastin, which are important in restoring matrix organization after stretching [31].

A previous study found that the decrease in gene expression occurs due to the degradation of collagen in ECM [32]. This degradation process is very important in the development, morphogenesis, remodeling, and repair of tissues. The main purpose of the remodeling process that occurs in the last stage of wound healing is to strengthen the tensile strength and obtain the normal structure of the tissue [33]. ECM degradation involves various types of proteases, but the most important type of protease is matrix metalloproteinase (MMP) [34]. MMPs and their inhibitors play important roles in wound healing by modifying the ECM, allowing cell movement and controlling ECM degradation and deposition, which are important for tissue remodeling. Based on previous findings, the treatment of fibroblast cells with PRP induced the expression of the MMP-13 (collagenase), along with MMP-3 and MMP-10 (stromelysin 1 and 2, respectively) and MMP-9 (gelatinase B) [31]. MMP-3 and MMP-10 were expressed to degrade collagen-III, proteoglycans, laminin, and fibronectin during wound healing [35]. This statement explains the cause of decreased collagen-III and fibronectin gene expression in the fibroblasts treated with CB-PL and PB-PL gels. In addition, MMP-13 is expressed by fibroblasts and is responsible for the remodeling of granulation tissue, modifying the function of myofibroblasts, inflammation, matrix degradation, and angiogenesis [36]. The gene expression of collagen-I and collagen-III decreased possibly due to the action of MMP-13 that degrades both types of collagens.

MMP-9 plays a role in supporting cell movement and the re-epithelialization process. MMP-9 also causes the degradation of fibronectin, which lead to the release of the active form of TGF-β that inhibits cell proliferation [34]. In addition, MMP-9 also causes the degradation of other ECM components such as collagen-I, collagen-III, and elastin [35]. These findings are consistent with the results in this study. On the other hand, the reduction in collagen gene expression may also be caused by PRP, which only activates fibroblast activity and collagen remodeling but not collagen production [31].

The wound healing process of HOMF treated with CB-PL and PB-PL gels occurred very quickly, and it was likely that there had been a remodeling process by MMPs that degraded collagen, elastin, and fibronectin on day 3 in this study. Nevertheless, the wound of the control group was still not completely closed and the cells were still in the proliferation stage, when the remodeling process has yet to occur. Therefore, the gene expression of collagen, elastin, and fibronectin in the control group was high due to the degradation of ECM by MMPs not yet occurring. 

### 2.5. PDGF-BB Released from PL Gels

**Figure 6:**
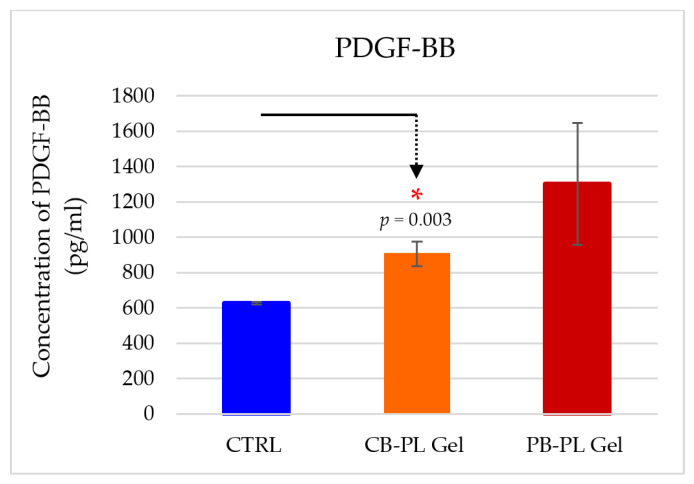
PDGF-BB released from gels of CB-PL and PB-PL and CTRL on day 3 of wound healing. The results were based on six independent tests (*n* = 6) and the means ± SEM were provided. The statistical significance of CB-PL gel compared to CTRL on day 3 was indicated by * *p* < 0.05.

PDGF-BB concentrations of CB-PL and PB-PL before gel polymerization were 35,250.71 ± 395.80 pg/mL and 53,015 ± 741.65 pg/mL, respectively, as published in our previous paper [37]. In this study, the protein level of PDFG-BB released from gels of CB-PL (905.48 ± 69.65 pg/mL) and PB-PL (1303.10 ± 343.96 pg/mL) was higher than the baseline of PDGF-BB in the control group (626.90 ± 8.01 pg/mL) on day 3 (Figure 6). There was no significant difference in PDGF-BB levels between both gels, although it showed a higher trend of PDGF release from PB-PL gel. This could be due to the fact that the PDGF-BB concentration of the isolated PB-PL was higher than that of CB-PL, although both were adjusted to the same number of platelets before undergoing freeze–thaw cycles. The use of different platelet derivatives or different PL preparation methods may also contribute to the different findings from other studies [16,38]. Noh and colleagues found that the secretion of PDGF-AB/BB peaked immediately after the low leukocytes’ PRP treatment and then decreased gradually over 10 days. PDGF-AB/BB enhanced the proliferation of fibroblasts and the production of other growth factors [39]. This study only measured the PDGF level at the end point (day 3) of the wound healing. The PDGF levels should be measured every day in order to obtain a clear pattern of PDGF release from PL gels in relation to the wound healing process in the future study. 

### 2.6. Limitation of Study and Future Studies

Future studies could investigate the degradation kinetics over longer periods of time to determine the optimal duration of treatment with PL gels. The effectiveness of PL gel should be compared with other wound healing treatments in the future study. The underlying wound healing mechanism of these PL gels remains elusive in this study. Hence, the downstream effects on tissue structure and function as a result of reduced ECM marker expressions should be investigated. The intracellular signaling cascades and MMP expression should be analyzed in the future study to elucidate the regulatory mechanism of PL gel in cell proliferation and migration during wound healing and remodeling. A comprehensive profiling of growth factors released from PL gels should be studied to understand their roles associated with the wound healing process. Besides that, the combination of biopolymers and PL to formulate a PL gel can be investigated to overcome the challenges of rapid gel degradation and the short half-life of growth factors. Additionally, the immunomodulation properties of PL gels in tissue regeneration should also be carried out in the future study. 

## 3. Conclusions

CB-PL gel and PB-PL gel accelerated the cell proliferation (148 ± 3% and 149 ± 3%), cell migration (110,651.46 ± 5094.11 μm^2^/day and 104,782.27 ± 4321.98 μm^2^/day), and wound closure (94.17 ± 1.77% and 92.75 ± 1.80%) in the wound healing assay, respectively. The gene expression of ECM markers (Col-I, Col-III, elastin, and fibronectin) was decreased after treatment with these two sources of PL. The PDGF-BB released from the PB-PL gels (1303.10 ± 343.96 pg/mL) was higher than that of CB-PL gels (905.48 ± 69.65 pg/mL), although their platelet counts were standardized. In addition, the slow CB-PL and PB-PL gel degradation rates (5.28 ± 0.72% and 9.55 ± 1.82%) give PL the potential for use as a topical gel in the future. Overall, this study provides valuable insights into the potential use of PL gels for wound healing, and future research could build upon these findings to further optimize treatment protocols. 

## 4. Materials and Methods

### 4.1. Tissue Collection and Handling

This study involved three types of human samples, which were two types of blood samples (cord blood and peripheral blood) and primary human mucosal fibroblasts (HOMFs). The umbilical cord blood was collected from six infant donors (*n* = 6) delivered at the Department of Obstetrics and Gynecology, Universiti Kebangsaan Malaysia Medical Centre (UKMMC). Peripheral blood was collected from six healthy adults (*n* = 6) by a phlebotomist at the Department of Anatomy, UKMMC. Six samples of primary human mucosal fibroblasts (*n* = 6) were isolated from the oral mucosal tissue that attached to the extracted wisdom tooth at the Oral Surgery Clinic or Oral Medicine Clinic, Faculty of Dentistry, Universiti Kebangsaan Malaysia (UKM).

#### 4.1.1. Cord Blood Collection and Processing

The cord blood was collected immediately using a needle into a sterile cord blood collection bag containing citrate-phosphate-dextrose-adenine anticoagulant (JMS, Singapore) by an obstetrician after the newborn delivery. Cord blood should only be collected in uncomplicated deliveries of infant donors from 34 weeks’ gestation or above according to the Fact-Netcord standard [40]. To comply with the standard, in this study, the cord blood was collected from the healthy new-born with no infectious diseases as indicated by the negative serology and laboratory findings for human immunodeficiency virus, hepatitis B virus, hypertension, and diabetes mellitus [41]. For cord blood processing, 10 mL of cord blood sample was aliquoted and centrifuged at 300× *g* for 25 min at room temperature (24 °C). The plasma layer was collected into a new centrifuge tube for the second centrifugation at 600× *g* for 5 min at room temperature. Following this, ⅔ of the upper part of the centrifuged plasma (platelet-low plasma) was removed and ⅓ of the centrifuged plasma (PRP) was retained [37]. A total of 1 mL of CB-PRP was sent to the Pathology unit, UKMMC to determine the platelet count using the SYSMEX Hematological Analyzer (SYSMEX, Tokyo, Japan). The platelet count of the remaining CB-PRP was standardized to 300 × 10^9^/L and maintained at −80 °C prior to preparing platelet lysate. 

#### 4.1.2. Peripheral Blood Collection and Processing

The adult donors must have a minimum age of 21 years old, ≥45 kg body weight, and adequate sleep (≥5 h) before blood withdrawal. A total of 30 mL of peripheral blood was collected from each donor into ten 3 mL K2EDTA tubes (BD, Franklin Lakes, NJ, USA). The blood sample was processed within 24 h after collection. For the first centrifugation, 10 mL of blood was transferred into a sterile centrifuge tube and spun at 100× *g* for 15 min. After that, the plasma layer was transferred into a new centrifuge tube and spun at 600× *g* for 5 min [42]. The following steps were similar to the CB processing procedure described above. The ⅔ upper part of the centrifuged plasma was removed and the remaining ⅓ of the centrifuged plasma (PRP) was collected. Subsequently, 1 mL of PB-PRP was sent to the Pathology unit, UKMMC for platelet count analysis. The platelet count of the remaining PB-PRP was standardized to 300 × 10^9^/L and maintained at −80 °C prior to preparing platelet lysate. 

#### 4.1.3. Isolation and Culture of Primary Human Oral Mucosal Fibroblasts

A 2 mm soft tissue specimen (excess normal tissues) was obtained either from wisdom tooth extraction or oral mucosal lesion biopsy. The patients were healthy subjects with no soft tissue lesions and did not consume drugs in the past six months. During the minor oral surgery procedure, some excess soft tissues were usually removed along with the extracted tooth. The excess tissue was scraped off from the extracted tooth root surface and kept in phosphate-buffered saline added with 1% antibiotic/antimycotic (Gibco-BRL, Waltham, MA, USA). The soft tissue specimen was then digested by 0.3% type I collagenase (Worthington Biochemical Corporation, Lakewood, NJ, USA) at a 37 °C shaker incubator for 15 min in order to detach the cells. The cells were cultured with Dulbecco’s Modified Eagle’s Medium/Nutrient Mixture F-12 (DMEM/F-12, Gibco-BRL, Paisley, Scotland, UK) enriched with 10% fetal bovine serum (FBS, Gibco-BRL), 1% antibiotic/antimycotic (Gibco-BRL, USA), 1% L-glutamine (Gibco, Tokyo, Japan), and 1% L-ascorbic acid (HmbG Chemicals, Hamburg, Germany) in a 5% CO_2_ incubator at 37 °C. The culture medium was replenished every 2–3 days until the cells reached 70–80% confluency. After that, the cells were treated with 2 mL of 0.05% trypsin-EDTA (Gibco-BRL, USA) at 37 °C for 5 min to detach the HOMFs [43]. The epithelial cells remained in the culture flask because they required a longer time to detach from the surface of flask. Cell characterization of HOMFs (passage 2) was carried out through cell morphology identification and gene expression of the fibroblast marker (vimentin). The data of cell characterization were published in our previous paper [37]. All assays were performed using HOMFs at passage 4.

#### 4.1.4. Platelet Lysate Gel Preparation

The total platelet counts of PRP obtained from cord blood (CB-PRP) and peripheral blood (PB-PRP) were 378 ± 59^9^/L and 486 ± 50^9^/L as published in our previous paper [37]. Both types of PRP were standardized to the platelet count of 300 × 10^9^ /L. PRP was submitted to three freeze–thaw cycles by alternating the temperature between −80 °C and 37 °C. The rapid change in temperature stimulates platelet lysis and the complete release of platelet-derived factors. Following the last freeze–thaw step, the PRP was centrifuged at 5000× *g* for 20 min at room temperature. The supernatant (platelet lysate) was separated from the pellet into a new sterile 50 mL centrifuge tube (SPL, Pocheon-si, Republic of Korea). All six samples of CB-PL and PB-PL were pooled separately and kept at −80 °C for further use. 

**Figure 7:**
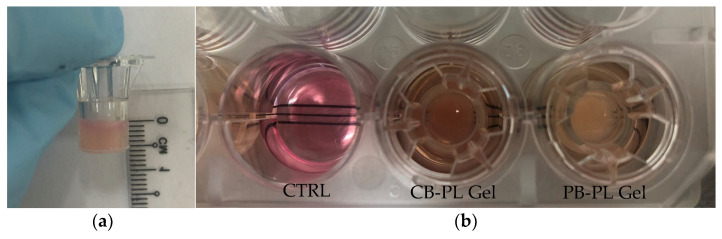
PL gels were formed with the addition of conditioned medium and CaCl_2_ in PL. (**a**) PL gel was collected for weight measurement on day 3 of the wound scratch assay and (**b**) PL gel with the culture insert was submerged in the 24-well plate for the wound scratch assay.

To prepare the 200 µL platelet lysate gel preparation, 160 µL of PL was added with 20 µL of conditioned medium from HOMF culture and 20 µL of 1 M calcium chloride solution (Merck, Darmstadt, Germany) in a 6.5 mm culture insert (Corning Inc., Kennebunk, ME, USA). The polymerization of gel took approximately 10–15 min at room temperature (Figure 7).

### 4.2. Alamar Blue Cell Proliferation Assay

HOMFs were seeded onto the 24-well plate (Corning Inc., Kennebunk, ME, USA) with a seeding density of 3 × 10^4^ cells per well and cultured in 1 mL of culture medium for 24 h. Next, the cells were washed with PBS before being replaced with serum-free medium containing DMEM/F-12, 1% antibiotic/antimycotic, 1% L-glutamine, and 1% L-ascorbic acid. The culture insert with CB-PL gel and PB-PL gel was submerged into the wells and incubated at 37 °C with 5% CO_2_ for three days. The control (CTRL) group was cells without PL gel. On day 3, the culture medium was discarded and replaced with 300 μL of Alamar blue solution (30 μL of Alamar blue stock reagent and 270 μL of serum-free medium). After 4 h of incubation, 100 μL of Alamar blue solution was transferred into three wells (triplicates) in a 96-well plate (SPL, Pocheon-si, Republic of Korea). The optical density was measured at the wavelengths of 570 nm and 600 nm using a microplate reader. The percentage of the Alamar blue reduction value was calculated according to the manufacturer’s manual. After that, the cell proliferation percentage was calculated using the following equation:

(1) $$\%\;\mathrm{Cell}\;\mathrm{Proliferation}=\left(\frac{{\%\;\mathrm{Reduction}\;\mathrm{Value}}_{\mathrm{treatment}}-{\%\;\mathrm{Reduction}\;\mathrm{Value}\;}_{\mathrm{CTRL}}}{\begin{array}{c} {\%\;\mathrm{Reduction}\;\mathrm{Value}\;}_{\mathrm{CTRL}} \end{array}}\right)\times 100\%$$

### 4.3. Wound Scratch Assay

**Figure 8:**
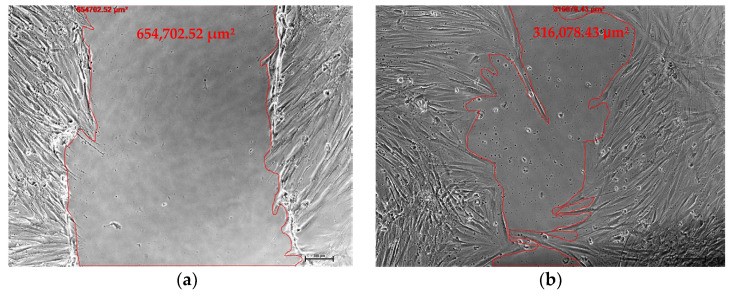
The wound area was demonstrated by the cell-free area within the scratch. The phase contrast micrographs of CTRL were taken on (**a**) day 0 and (**b**) day 2. Each photo was calibrated with 100× magnification. The surface area of the wound was measured in units of μm^2^ using Axiovision software.

HOMFs were cultured until 100% confluent in the 24-well plate (Corning Inc., Kennebunk, ME, USA) with 3 grid lines (3 mm gaps) (Figure 7b). Two scratches (12 mm length) were made on the confluent monolayer cells using a sterile white pipette tip in order to simulate in vivo wounds. After that, the culture medium and cell debris were removed and rinsed with 1 mL of PBS. Subsequently, the cells were replenished with 1 mL of serum-free medium. The culture inserts with CB-PL gel and PB-PL gel were submerged into the wells, while the CTRL was scratched monolayer cells without PL gel. The sequential photos were taken from 2 spots between two grid lines using Olympus Inverted Microscope Models IX71 and Cell B software (Olympus, Tokyo, Japan) in each group on days 0, 1, 2, and 3. After taking the photos on day 3, the culture medium of each group was harvested for PDGF-BB measurement. The cells were lysed and preserved with 1 mL of Trizol reagent (Invitrogen, Life Technologies, Carlsbad, CA, USA) for qRT-PCR. On the other hand, the culture insert with PL gel was collected for weight measurement. For wound area measurement, the cell-free area within the scratch was measured using the outline feature in the Axiovision software version 18.4 according to the manufacturer’s manual (Figure 8). The wound areas from 2 spots were measured and averaged for each group. 

The wound closure percentage was determined using the following equation:

(2) $$\%\;\mathrm{Wound}\;\mathrm{Closure}=\left(\frac{A_{t\;=\;0d}-A_{t\;=\;∆d}}{\begin{array}{c} A_{t\;=\;0d} \end{array}}\right)\times 100\%$$

*A*_*t* = 0__d_ = Area of wound measured immediately after scratching (*t* = 0 d);

*A_t = _*_Δd_ = Area of wound measured at d days after scratching.

The cell migration rate was calculated using the following equation:

(3) $$\mathrm{Cell}\;\mathrm{Migration}\;\mathrm{Rate}\;=\left(\frac{A_{t\;=\;0d}-A_{t\;=\;∆d}}{\mathrm{day}}\right)$$

*A*_*t* = 0__d_ = Area of wound measured immediately after scratching (*t* = 0 d);

*A_t = _*_Δd_ = Area of wound measured at d days after scratching.

### 4.4. Gel Degradation Measurement 

The gels in the culture insert were submerged in the serum-free culture media at pH 7.22 and incubated at 37 °C with 5% CO_2_ for three days. The weight of the culture insert with PL gel was measured using a digital balance (Sartorius, Göttingen, Germany) on days 0 and 3. Gel degradation was represented by the weight loss of gel due to the gradual release of PL gel content [27]. The percentage of gel degradation was determined using the following equation:

(4) $$\mathrm{Percentage}\;\mathrm{of}\;\mathrm{Gel}\;\mathrm{Degradation}=\left(\frac{{\mathrm{Gel}\;\mathrm{Weight}}_{\mathrm{day}0}-{\mathrm{Gel}\;\mathrm{Weight}}_{\mathrm{day}3}}{\begin{array}{c} {\mathrm{Gel}\;\mathrm{Weight}}_{\mathrm{day}0} \end{array}}\right)\times 100\%$$

### 4.5. Two-Step Quantitative Reverse Transcriptase-Polymerase Chain Reaction (RT-qPCR)

The total ribonucleic acid (RNA) extraction was carried out according to the manufacturer’s manual. Briefly, 1 mL of cell lysate was added with 200 μL of chloroform and centrifuged at 12,000 rpm for 15 min in order to separate the aqueous and organic phases. The aqueous phase containing RNA was transferred into a sterile microcentrifuge tube (Eppendorf, Hamburg, Germany) and precipitated with 500 μL of isopropanol and 5 μL of a polyacryl carrier (Molecular Research Center, Cincinnati, OH, USA). After that, the RNA pellet was rinsed with 1 mL of 75% ethanol and resuspended with 10 μL of DNAse and RNAse-free distilled water (Invitrogen, Carlsbad, CA, USA). The RNA was quantified using a Nanodrop ND-100 spectrophotometer (Nanodrop Technologies, Wilmington, Delaware, DE, USA). Following this, complementary deoxyribonucleic acids (cDNA) were synthesized from 100 ng of total RNA using the SuperScript™ IV First-Strand Synthesis SuperMix kit (Invitrogen, USA), according to the manufacturer’s manual. For cDNA synthesis, the protocol of reverse transcription involved primer annealing (10 min at 23 °C), reverse transcription (60 min at 50 °C), and termination of the reaction (5 min at 85 °C). Table 2 shows the forward and reverse primer sequences of 4 target genes (collagen-I, collagen-III, elastin, and fibronectin) and the housekeeping gene (gIyceraIdehyde-3-phosphate dehydrogenase, GAPDH) [38]. qPCR was performed using the Luna^®^ Universal qPCR master mix (NEB, Ipswich, MA, USA) in the Bio-Rad real-time thermal cycler CFX96^TM^ (Bio-Rad, Hercules, CA, USA). The qPCR protocol involved the activation of Taq DNA polymerase at 95 °C for 3 min, 40 cycles of PCR amplification at 95 °C for 10 s and 61 °C for 30 s, and melt curve analysis. For data analysis, the relative quantification of mRNA levels of a target gene to the housekeeping gene was calculated using the following equation:

(5) $$\mathrm{Relative}\;\mathrm{expression}\;\mathrm{ratio}\;=2^{-\left(∆\mathrm{CTtarget}\;\mathrm{gene}-∆\mathrm{CTGAPDH}\right)}$$

∆CT target gene = threshold cycle value of target gene

∆CT GAPDH = threshold cycle value of housekeeping gene

### 4.6. PDGF-BB Measurement via Enzyme-Linked Immunosorbent Assay (ELISA)

The protein level of PDGF-BB was quantified using an ELISA kit (Biorbyt, Cambridge, UK) based on the manufacturer’s instructions. Briefly, the 96-well microplates were pre-coated with a target-specific antibody for human PDGF-BB protein before incubation with 100 µL of standards or samples at room temperature for 90 min to allow PDGF-BB antigen binding to the immobilized antibody. Then, the biotinylated anti-human PDGF-BB antibody was added. The unbound biotinylated antibody was washed away and Horseradish-Peroxidase-conjugated streptavidin was added to the wells. Subsequently, the wells were washed again and added with TMB substrate solution. The stop solution was added before the optical density of the product was measured at a 450 nm wavelength using an ELISA microplate reader.

### 4.7. Statistical Analysis

Statistical analyses were performed using Statistical Package for the Social Sciences (SPSS) version 28.0 software (IBM Corp, Armonk, New York, NY, USA). Parametric tests were carried out for normally distributed data. One-way analysis of variance (ANOVA) with Tukey’s HSD tests were used to determine the statistical significance between groups for the parameters, i.e., cell proliferation, wound closure, cell migration, qRT-PCR, and PDGF-BB levels. The independent *t*-tests were carried out to compare the means between two groups for data of gel degradation. The statistical significance was indicated by *p* < 0.05.

## Figures and Tables

**Table 1:** Relative mRNA expression of ECM markers during wound healing.

ECM Marker	Group	Relative mRNA Expression to GAPDH (Mean ± SEM)
Col. I	CTRL	1.47 ± 0.35
	CB-PL gel	0.13 ± 0.07
	PB-PL gel	0.09 ± 0.06
Col. III	CTRL	0.64 ± 0.13
	CB-PL gel	0.09 ± 0.03
	PB-PL gel	0.05 ± 0.02
Elastin	CTRL	0.0135 ± 0.0042
	CB-PL gel	0.0018 ± 0.0010
	PB-PL gel	0.0012 ± 0.0007
Fibronectin	CTRL	2.28 ± 0.45
	CB-PL gel	1.47 ± 0.35
	PB-PL gel	0.87 ± 0.22

**Table 2:** Forward and reverse primer sequences of ECM markers and housekeeping gene.

Gene	GenBank Accession Number	Primer Sequence (5′ to 3′)
GAPDH	NM_002046.5	F: CAATGACCCCTTCATTGACC
		R: TTGATTTTGGAGGGATCTCG
Collagen-I	NM_000088.3	F: GTGCTAAAGGTGCCAATGGT
		R: ACCAGGTTCACCGCTGTTAC
Collagen-III	NM_000090.3	F: CCAGGAGCTAACGGTCTCAG
		R: CAGGGTTTCCATCTCTTCCA
Elastin	NM_000501.4	F: GGTGGCTTAGGAGTGTCTGC
		R: CCAGCAAAAGCTCCACCTAC
Fibronectin	NM_212482.2	F: AAAATGGCCAGATGATGAGC
		R: TGGCACCGAGATATTCCTTC

## Data Availability

Not applicable.
